# Implementation of statutory oral health examinations among Finnish preschool children: a register-based pilot study

**DOI:** 10.2340/aos.v84.44871

**Published:** 2025-11-25

**Authors:** Anna-Maria Pelkonen, Päivi Rajavaara, Hannu Vähänikkilä, Vuokko Anttonen, Marja-Liisa Laitala

**Affiliations:** a Research Unit of Population Health, University of Oulu, Oulu, Finland; b The Wellbeing Services County of North Ostrobothnia, Pohde, Finland; c Medical Research Center Oulu, Oulu University Hospital and University of Oulu, Oulu, Finland; d Northern Finland Birth Cohorts, Arctic Biobank, Infrastructure for Population Studies, Faculty of Medicine, University of Oulu, Oulu, Finland

**Keywords:** Statutory oral health care, Health Care Act, preschool-aged, oral health examination, cooperation

## Abstract

**Objective:**

The purpose of statutory oral health care services based on the Health Care Act (implemented 01 May 2011) and Decree (implemented 06 April 2011) is to provide equal services nationwide for all children. The aim of this register-based study was to explore the implementation and content (multiprofessional co-operation, absenteeism and the need for family’s special support) of statutory oral health examinations and screenings among a group of Finnish preschool children.

**Materials and methods:**

The study group consisted of the medical records of Finnish children in the City of Oulu from three different age groups (born in 2014–2018, *n* = 2,023–2,456). In this pilot study, data on dental examinations/screenings and missed appointments and their reasons of 206 randomly selected preschool-aged were collected from patient records in oral and public child health clinics during March 2022 to July 2022. Referrals within oral health care, along with the occupations of those referred to, were registered. Chi-squared or Fisher’s Exact Test was used to evaluate differences between three age groups.

**Results:**

Across different age groups, 8.7% – 21.4% of children dropped out of statutory oral health care services. Dentists performed only a small proportion of oral health examinations for preschool-aged children (3.1%). Co-operation between oral health professionals was scarce. They rarely solved the reasons for missed appointments of dental care visits (5.1%). No referrals to Child Protection Services were made from oral health care.

**Conclusion:**

In this group of preschool children, implementation of Health Care Act and Decree was only partially completed. Absenteeism from statutory oral health care, addressing the need for Child Protection Services, and lack of multiprofessional co-operation seem to be causes for concern. Further research and attention to this topic is necessary.

## Background

Every child has the right to good health, including oral health [1]. To support parents and caregivers, dental professionals are responsible for ensuring the realisation of children’s rights and, in particular, monitoring implementation of laws in their field of expertise. Ensuring access to dental care for all children is important to prevent oral health disparities [2].

Free health and oral health care for children and young people under the age of 18 is regulated by Finnish laws and decrees [3, 4]. Statutory oral health care services are meant to even out health inequalities and provide equal services nationwide. Children under school age are entitled to at least three oral health examinations and if needed, additionally according to their individual needs. According to the statistics of the Finnish Institute of Health and Welfare in 2023 [5], 64.4% of Finnish 5-year-olds and 67.9% of children living in Northern Ostrobothnia had undergone an oral health examination/screening. The Decree (338/2011 implemented 06 April 2011) related to the Health Care Act (1326/2010, implemented 01 May 2011) emphasises the responsibility of oral health care professionals to identify and also to provide special services to children at risk. Every health care professional is also obliged to make a referral to Child Protection Services if there is suspicion of child abuse [3]. One form of child abuse is neglect of dental care, in which case the child’s right to receive appropriate health care has not been fulfilled.

The Finnish maternity and child health clinic system also provides a wide range of services for children, and offers the potential for comprehensive, collaborative care provided by different health professionals, including oral health professionals. In addition to other health-related habits, public health nurses at maternity clinics are instructed to evaluate a child’s oral health and related factors, and refer the child to oral health care if needed [6]. Oral health care professionals should not only cooperate with each other, but also with other professionals, that is, public health nurses at the maternity clinics [7] as well as Child Protection Services. According to Finnish law, only dentists can set diagnoses. Dental nurses and oral hygienists are allowed to perform oral screenings and record findings in patient files independently. If any signs of oral diseases or oral health-related risk factors are detected, paediatric patients should be referred further to a dentist. Enforcement of the implementation of statutory oral health examinations by Finnish authorities is not systematic and to our knowledge, research considering this topic has not been published.

The aim of this register-based pilot study was to investigate implementation of statutory oral health examinations of preschool-aged children in the city of Oulu, Finland. In addition, involvement of different professionals as well as practices related to missed appointments were targets of interest. Again, it was investigated whether a child’s and family’s need for special support and possible need for contacting Child Protection Services were recognised by oral health professionals as emphasised in the law.

## Materials and methods

### Data collection

The STROBE Checklist was used to design the study [8]. This data-based study was based on the medical records of 206 randomly selected children under 7 years old, living in the city of Oulu, Finland, during the entire study period, 2014–2020. Data of children in the age group of 5–6 years (children who were born in 2014), in the age group of 3–4-years (children who were born in 2016), and in the age group of 1–2-years (children who were born in 2018) were randomly collected by using a number generator (Calculator.net) between March 2022 to July 2022. The data of the source population (all children who were born in 2014, 2016, and 2018, and the total number of children 2,456, 2,249, and 2,023, respectively) were provided by official statistics of the City of Oulu.

The total number of children was 206 (born in 2014, 2016, or 2018) who had a statutory oral health examination or screening at the age of 1–2-years. Of those born in 2014 or 2016, had a statutory oral health examination at the age of 3–4-years (*n* = 142) and those born in 2014 had had a statutory oral health examination or screening at the age of 5–6-years (*n* = 69). Total number of examinations at certain age levels at public child health clinics was 411 (a child could have had 1–3 examinations during the follow-up period). Seven children did not have an examination at public child health clinic.

Children from urban and rural areas of Oulu and also those who did not have any contact or visit at oral health services were included. Data collection continued until at least 54 children with statutory examinations from each age group were obtained [9].

The child’s year of birth and gender (*f/m/othe*r) were registered. The children’s dental care visits (*number of oral health examinations/screenings*) and the profession of the person performing the examination/screening (*dentist/oral hygienist/dental nurse*) were registered. The data of missed appointments (*n*), referrals (*n, to whom*), as well as detecting child neglect and sending notices to Child Protection Services (*written entry in patient records*), were collected from the patient records in the oral health care system for all children. Information on missed appointments (*n*) in public child health clinics and referrals (i.e., public health nurses recommended to make an appointment) to oral health care (*n*) of all included children was also collected from the records of public health nurses. The family-related risk indicators (*parental exhaustion, sleeping or behavioural problems of the child, death or grief in the family, need for support from social services or child living in out-of-home care, guardian’s unemployment or self-reported problems with mental health, divorce of parents or problems in the parents’ relationship*) were also registered at least once (*yes/no*). No one was excluded from the study population due to problems in general health or physical or mental disabilities. Children who had moved to or from the study location during the study period were excluded from the study.

### Data and statistical analysis

Power calculations were performed to determine the sample sizes assuming that the difference in decay between the different groups is *d* = 1.2–0.7 = 0.5. With the significance level alpha = 0.95 and power (1-Beta) = 0.8, the sample size was 54 per age group.

The results were presented as distributions and frequencies as well as graphically. Chi-squared and Fisher’s Exact Test were used to evaluate differences between referrals from a public child health clinic to a public dental clinic for all age groups. The differences were considered statistically significant if *p* < 0.05. IBM SPSS Statistics for Windows, Version 29.0 (Armonk, NY: IBM Corp) was used for the analyses.

### Ethical considerations

Permission for the study was granted by the City of Oulu, on 19 November 2021 (OUKA/12798/07.01.04.02/2021). In accordance with Finnish legislation (Data Protection Act 1050/2018), no statement from the regional medical research ethics committee of the Wellbeing Services County of North Ostrobothnia was necessary. Participant anonymity was maintained throughout the study by assigning ID numbers, with the key held by the first author.

## Results

### Completed oral health examinations and screenings

Statutory oral health examinations were carried out for 78.6% of 1–2-year-olds, 81.7% of 3–4-year-olds, and 91.3% of 5–6-year-olds in this study population. Dentists seldom performed oral health examinations of children under school age. For 1–2- and 3–4-year-olds, oral health screenings were most often performed by a dental nurse. Among 5–6-year-olds, the most common professional group performing examinations were oral hygienists. Children who were referred to an oral health examination at the age of 1–2 years by a public child health clinic employee were more likely than those without referral (89.7% vs. 64.4%) to attend the oral examination at that age level (*p* < 0.001) (Table 1).

**Table 1:** Information about oral health care screenings/examinations in different age groups.

Variable	Oral health screening/examination *n* (%)		
	1–2-years (*n* = 206)	3–4-years (*n* = 142)	5–6-years (*n* = 69)
**Completed oral health screening or examination done by**	162 (78.6)	116 (81.7)	63 (91.3)
a dentist	3 (1.9)	3 (2.6)	3 (4.8)
an oral hygienist	34 (20.9)	28 (24.1)	45 (71.4)
a dental nurse	125 (77.2)	85 (73.3)	15 (23.8)
**Referred from a public child health clinic to a public dental clinic**	116 (56.6)	55 (40.4)	18 (26.1)
Examination done	104 (89.7) *	49 (89.1)	17 (94.4)
Examination not done	12 (10.3)	6 (10.9)	1 (5.6)

* *p* < 0.001..

### Collaboration between dental professional groups

**Figure 1:**
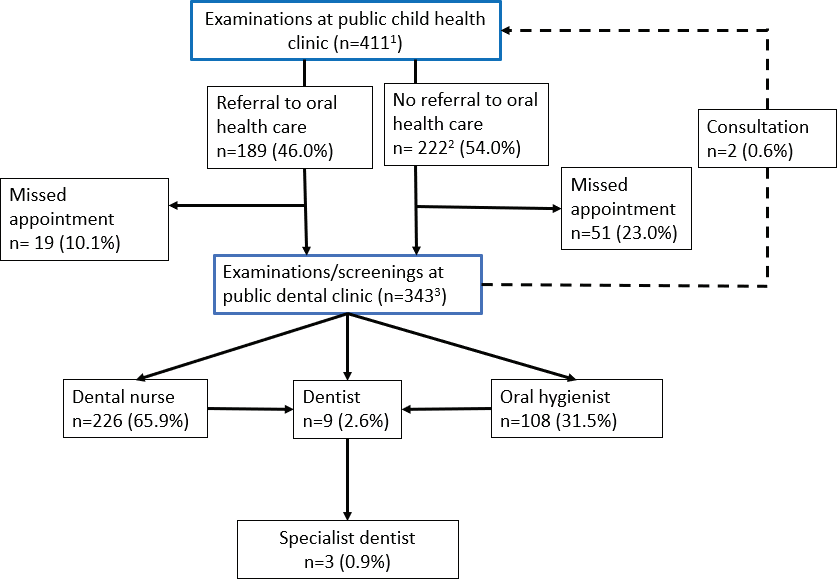
Treatment paths, consultations, and referrals in the oral health care of preschool-aged children from 2014 to 2020. ^1^Seven children did not have an examination at public child health clinic. ^2^An appointment for examinations/screening was booked by a public dental clinic or the care giver. ^3^Two children were examined by both a dental nurse and/or oral hygienist and a dentist

Children were referred to the dentist by oral hygienists or dental nurses as follows: 5.5% of the 1–2-year-olds, 6.8% of the 3–4-year-olds, and 22.2% of the 5–6-year-olds. The most common cause for a referral to a dentist was dental caries. Not all children were evaluated by a dentist, even when an oral hygienist or a dental nurse detected caries lesions (initial or dentinal). In the case of detected carious lesions, 42.9%, 66.7%, and 36.8% of children, depending on the age group, did not receive an oral examination by a dentist. Three children from the entire study material were referred to a paediatric specialist dentist (0.9%). Less than half of the children from the public child health clinic were referred for an oral health examination (Figure 1).

### Detecting neglect and referrals to child protection services

A statistically significant association was found between missed appointments at the public child health clinic and the need for child protection services at 1–2 years of age (*p* = 0.002). Referrals to Child Protection Services submitted by a public child health clinic, day care or some other parties were recorded in the health records of 7.3% of the children. No referrals to Child Protection Services were made by oral health care professionals. One child had a record of suspected negligence, but no indication of further action was determined.

### Missed appointments in statutory oral health services

In this study, all participants had at least one oral health care appointment in addition to public child health care clinic visits. The highest dropout rate from the statutory oral health care was found among the 1–2-year-olds. At that age, a total of 21.4% of the children born in 2014, 2016, or 2018 did not participate in oral health examinations or screenings. The respective proportion of the 3–4-year-olds was 19.0% and of the 5–6-year-olds 10.1%. The most common causes discovered in the patient records for not participating in the statutory oral health examination or screening were the processes in the organisation: parents were not referred from the public child health clinic for an oral health examination, or a visit to oral health care was only recommended rather than directly scheduling an appointment on behalf of the parents (Table 2).

**Table 2:** Causes of and responses to missed oral health screenings/examinations in statutory oral health care services.

Variable	Proportions of causes for missed appointments in different age groups *n* (%)		
	1–2-years (*n* = 44)	3–4-years (*n* = 27)	5–6-years (*n* = 7)
**Care giver-based reasons**			
No-show to booked appointment	14 (31.8)	1 (3.7)	2 (28.6)
Appointment not booked by care givers	2 (4.5)	14 (51.9)	1 (14.3)
Care giver refused to book appointment	1 (2.3)		
Organisation-based reason*	27 (61.4)	12 (44.4)	4 (57.1)
**Response to missed dental appointment by organisation**	1 (2.3)	2 (7.4)	1 (14.3)

* Oral health professionals did not make an appointment for the child and/or the child was not referred from a public child health clinic for dental care.

Some of the children who were examined missed several statutory examination visits. Of the 3–4-year-old children and of the 5–6-year-old children, 9.2% and 10.1% missed more than one oral health examination/screening, respectively. Of these dropouts, 45.4% had a family-related or dental attendance risk indicator in patient document entries. Some of the children (28.5%) missed both public child health clinic visits and oral screening/examinations at least once during their age group. Depending on the age group, 18.8% – 21.8% of the children missed one or more appointments at public child health clinics. Oral health professionals intervened in missed statutory visits only in a few cases. Absenteeism was addressed in 5.1% of cases (Table 2).

## Discussion

This study found shortcomings in the implementation of the Health Care Act in the City of Oulu for children. Children’s right to adequate health services in terms of oral health care was not realised for everyone, particularly for those at risk, as mandated by law. Collaboration between oral health care professionals, maternity and child health clinic services, or child welfare services was not adequate. In this study, it seems that the health care system was not able to provide comprehensive services to all paediatric patients, nor effectively enough to prevent the polarisation of oral and dental health disparities.

The results of this study show that not all preschool-aged children received public oral health care services according to the law. It is noteworthy that a major proportion of missed statutory oral health examinations were due to organisational factors, suggesting that public oral health care may be neglecting its duty to provide primary health care services for all children. To prevent the polarisation of oral health problems among children, special attention should be paid to this issue. Although the proportion of those who got statutory oral health examination was acceptable and in line with national records [5], individual needs were not properly recognised. According to the registers, using private oral health care services was not mentioned as a reason for missed appointments. When compared internationally, oral health service attendance in this study among preschoolers seems to be higher than average [10–13]. However, straightforward comparisons cannot be made because the health care systems are not comparable. Obligations of society with regard to paediatric dental care differ, as in some countries it is totally dependent on parental activity. Implementation of oral health examinations/screenings was least successful in the youngest age group. Therefore, oral health care services were more accessible to children of older age groups. This is in line with a recent review by Goswami et al. [14], which concluded that non-utilisation of dental health services tends to be higher in younger children.

In Finland, dental nurses and oral hygienists perform a majority of the oral health screenings of children below school age [15, 16], which indicates changes in operating models and division of labour between oral health professionals. The dentists here performed only a small minority of the examinations. Unfortunately, a group of children who showed signs of dental caries lesions, based on the dental nurse’s or oral hygienist’s records, were not referred to a dentist. One reason why children are not being referred to dental care could be perception that deciduous teeth do not need restorative treatment due to exfoliation. Additionally, caries diagnostics is challenging, and caries lesions can be undetected. This finding emphasises that there is still a need to update good clinical practices. Delays in obtaining a more accurate diagnosis and appropriate treatment may have serious consequences, such as pain, oral infections, and even the need for dental general anaesthesia, which could have been avoided through preventive measures and minimally invasive treatment.

According to the registers, about 7.0% of children required Child Protection Services, but none of them were detected and referred by oral health care personnel. In 2023, child welfare notifications were made of 5.9% of 0–2-year-old children and 7.9% of 3–6-year-old children in Finland [17], which align with the results of this study. Public child health clinic professionals were more aware than dental professionals of the need for special support or child welfare for the child and family. According to a recent study, dental professionals in Finland are uncertain about observations regarding child abuse and neglect [18], which is in line with this study. This raises concerns about whether the situation of disadvantaged families will deteriorate further when the need for support is neglected. Dental professionals meet children regularly if children have dental problems, which often is the case in families with challenges. Therefore, dental personnel have a unique vantage point, and awareness education on this topic is necessary [19].

Working with at-risk families requires seamless co-operation between different occupational groups. This study showed that there is a need for improvement in the co-operation between oral health care professionals. In addition to carrying out statutory oral health examinations and screenings, the challenge was also to utilise co-operation among all professional groups (e.g., maternity and child health clinic services). Previous studies found referrals by health care professionals to oral health services [20, 21], and collaboration between all health care professionals increased young children’s access to dental care [20]. This is in line with the results of this study. Verlinden et al. [22] reported that referrals also decreased caries experience when combined with a preventive first dental visit. Regular oral health examinations based on a patient-specific risk assessment have been shown to reduce the occurrence of dental caries [23]. Identifying patients at risk enables allocation of resources to those individuals most in need and who are most likely to benefit from treatment [24, 25].

Based on this study, missing appointments for oral health services were not addressed according to the law, as professionals only responded in 5.1% of the cases. The reasons for the low percentage might be the lack of organisational guidance and established practices. The responsibility of professionals in addressing absenteeism should be more emphasised and education on this issue seems to be inevitable for all oral health professionals. Also, in a previous Finnish survey [16] deficiencies were found in the investigation of the underlying reasons for missed appointments of children in dental care visits. Parents’ willingness to provide access to dental care services for their children varies. There are several background factors that influence outcomes, for example maternal oral health knowledge and social support [26], which, however, were not studied here. It is also known that demanding treatments like extractions may be associated with nonattendance in dental care [27]. Here, we found that children facing challenges in family life tended to miss appointments in dental care. Resources should be directed towards children known to have problems with oral health but missing dental care appointments, and their attendance should be monitored closely.

Oral health inequalities tend to persist, and the targeting of suitable preventive efforts for at-risk individuals should be considered when making health care policies [28]. The focus should be on the structural determinants of oral health inequalities, such as economic drivers, rather than solely focusing on changing oral health behaviours [29]. The American Academy of Pediatric Dentistry has made a policy [30] for recognising the importance of the social determinants of oral health in children. This policy emphasises interdisciplinary approaches to improve children’s oral health, taking into account social determinants of chronic diseases, and encourages oral health professionals to refer patients to support services when needed. Social determinants, however, were not studied here.

Here, register data as well as individual electronic patient records from different sources of the study population were available. In Finland, researchers can utilise register data related to oral health, which is collected comprehensively. This allows for the randomisation of the participants, and thus the results can be generalised to the under school-aged population living in Northern Finland. The participation of 5-year-old children in oral health examinations in this study was 72.4% and according to data from the quality register [5] 67.9% in North Ostrobothnia and 64.4% in Finland as a whole. The number of subjects in some groups was rather small, and participation rate remained low, which can be considered as a limitation. No previous scientific evidence, for example, absenteeism and referrals to oral health care from public child health clinics, was available for power calculations which were performed using dental decay as an outcome variable. The numbers of completed oral health screenings/examinations performed by dental hygienist/oral nurse or dentist in the oldest age group (5–6-years old) did not differ between the source population and the study sample significantly. However, generalisability of the results in this pilot study requires further research with larger study populations. Although Finnish oral health care records are considered highly reliable, and all dental visits and treatment procedures are registered [31], some details may had been missed, for example, reasons for missed appointments. Possible inadequate registering must be kept in mind as a limitation in register-based studies. Further, only limited number of factors related to the implementation of the Health Care Act and Decree were investigated.

The purpose of the Health Care Act and Decree is to guarantee oral health services according to a child’s and family’s needs. This study showed that the system failed in this for some children, collaboration between professionals was scarce, causes for missed appointments were seldom registered, and no referrals were made to Child Protection Services. The reasons for all this were not studied here but should be studied in future. Practices and situation in the family are essential for targeting services to those in need to prevent and manage oral diseases. In the future, work processes should be further developed and refined to remove obstacles to effective collaboration between dental professionals and between dental and other health and social service professionals. The purpose of the Health Care Act and its related decree could thus be better achieved.

## Declaration

### Availability of data and materials

The data that support the findings of this study are not openly available due to reasons of sensitivity and are available from the corresponding author upon reasonable request. Data are located in controlled access data storage at University of Oulu.
